# A method for independent component graph analysis of resting‐state fMRI

**DOI:** 10.1002/brb3.626

**Published:** 2017-02-16

**Authors:** Demetrius Ribeiro de Paula, Erik Ziegler, Pubuditha M. Abeyasinghe, Tushar K. Das, Carlo Cavaliere, Marco Aiello, Lizette Heine, Carol di Perri, Athena Demertzi, Quentin Noirhomme, Vanessa Charland‐Verville, Audrey Vanhaudenhuyse, Johan Stender, Francisco Gomez, Jean‐Flory L. Tshibanda, Steven Laureys, Adrian M. Owen, Andrea Soddu

**Affiliations:** ^1^ Department of Physics & AstronomyBrain & Mind InstituteWestern UniversityLondonONCanada; ^2^ Coma Science GroupGIGA ResearchUniversité et Centre Hospitalier Universitaire de LiègeLiègeBelgium; ^3^ IRCCS SDN, Istituto di Ricerca Diagnostica e NucleareNaplesItaly; ^4^ Brain and Spine Institute(ICM)HôpitalPitié‐SalpêtrièreParisFrance; ^5^ Department of Algology and Palliative CareUniversité de LiègeLiegeBelgium; ^6^ Department of Neuroscience and PharmacologyUniversity of CopenhagenCopenhagenDenmark; ^7^ Department of MathematicsUniversidad Nacional de Colombia sede BogotáBogotáColombia; ^8^ Department of NeurologyUniversité de LiègeLiègeBelgium; ^9^ Department of PsychologyBrain & Mind InstituteWestern UniversityLondonONCanada

**Keywords:** BOLD fMRI, graph theory, independent component analysis, resting state

## Abstract

**Introduction:**

Independent component analysis (ICA) has been extensively used for reducing task‐free BOLD fMRI recordings into spatial maps and their associated time‐courses. The spatially identified independent components can be considered as intrinsic connectivity networks (ICNs) of non‐contiguous regions. To date, the spatial patterns of the networks have been analyzed with techniques developed for volumetric data.

**Objective:**

Here, we detail a graph building technique that allows these ICNs to be analyzed with graph theory.

**Methods:**

First, ICA was performed at the single‐subject level in 15 healthy volunteers using a 3T MRI scanner. The identification of nine networks was performed by a multiple‐template matching procedure and a subsequent component classification based on the network “neuronal” properties. Second, for each of the identified networks, the nodes were defined as 1,015 anatomically parcellated regions. Third, between‐node functional connectivity was established by building edge weights for each networks. Group‐level graph analysis was finally performed for each network and compared to the classical network.

**Results:**

Network graph comparison between the classically constructed network and the nine networks showed significant differences in the auditory and visual medial networks with regard to the average degree and the number of edges, while the visual lateral network showed a significant difference in the small‐worldness.

**Conclusions:**

This novel approach permits us to take advantage of the well‐recognized power of ICA in BOLD signal decomposition and, at the same time, to make use of well‐established graph measures to evaluate connectivity differences. Moreover, by providing a graph for each separate network, it can offer the possibility to extract graph measures in a specific way for each network. This increased specificity could be relevant for studying pathological brain activity or altered states of consciousness as induced by anesthesia or sleep, where specific networks are known to be altered in different strength.

## 1 Introduction

The evaluation of functional connectivity from resting‐state fMRI data is broadly based on two families of analytical methods. Seed‐based correlation analysis estimates the relationship between a predefined region (the “seed”) and all other voxels around the brain (Biswal, Yetkin, Haughton, & Hyde, 1995; Fox & Raichle, 2007; Fox et al., 2005). A commonly employed alternative is independent component analysis (ICA; Beckmann, DeLuca, Devlin, & Smith, 2005; Damoiseaux et al., 2006; Esposito et al., 2008; Hyvärinen, Karhunen, & Oja, 2001), a data‐driven approach to decompose whole‐brain BOLD signal into a number of contributing volumetric spatial maps and their associated time‐courses, such that the spatial independence of the components is maximized. ICA has proven to be an effective and robust tool for the isolation of low‐frequency resting‐state patterns from data acquired at various spatial and temporal resolutions (De Luca, Beckmann, De Stefano, Matthews, & Smith, 2006). The ICA‐generated volumetric maps are generally reported as *z*‐scores in order to show the contribution of each component's time‐course to the BOLD signal in each voxel. These maps are then commonly analyzed in a voxel‐wise manner in order to show between or within‐group connectivity differences (Greicius, Srivastava, Reiss, & Menon, 2004). The number of components is generally user‐defined and many of the resulting components may be due to non‐neuronal activity (such as cardiovascular signal, eye movements, muscle activity in the vicinity of the head and head movement). Classically 10 functional networks can be reliably identified from ICA (Beckmann et al., 2005; Damoiseaux et al., 2006; De Luca et al., 2006; Fox & Raichle, 2007) by decomposing the signal and to separate neuronal from non‐neuronal components. Apart from voxel‐wise statistics, graph theory could be applied to the functional networks which provides tools for analysis of network topography and connectivity (Rubinov & Sporns, 2010). Functional and structural connectome analysis has become increasingly common in neuroscience. In these methods, the brain is defined abstractly as a network of nodes with edges connecting them. Nodes are often anatomically defined regions, whereas edges typically carry weights describing the correlation, similarity, or degree of connectivity between nodes. Adjacency graphs, in which the edge's existence is binary and carry no representative weight, are also common, as data‐driven approaches for brain parcellation (Bullmore & Bassett, 2011). Usually, a weighted graph *W* is created by calculating a connectivity measure between every pair of regions; subsequently, the meaningful properties of the graph are carried out by network analysis methods, such as summary statistics (e.g., degree, small‐worldness) and permutation testing (Zalesky, Fornito, & Bullmore, 2010). To our knowledge, no graph theory approach has been developed to evaluate the organization properties within brain networks derived from ICA. A graphical method permits to extract properties of a region of interest in the context of the full network. A comparison of graphical properties goes far beyond the comparison of the *z* values, as commonly done with the comparison of IC spatial maps voxel by voxel, where each voxel is treated as a separate entity and not considered in the context of the full brain spatial map. The method we present here incorporates pieces of both families of methods and it is a generalization to the entire brain of a previously introduced approach restricted to the default mode network (DMN) to study functional connectivity changes in patients with disorders of consciousness (Soddu et al., 2012). Following, this approach has been also applied in patients suffering from tinnitus focusing on the auditory network (Maudoux et al., 2012a, 2012b). We do believe that our method, for the very first time, permits a comparison of IC spatial maps using the advantage of graph theory.

## 2 Methods

In short, we used ICA and machine learning classification to isolate a set of neuronal components (Demertzi et al., 2014), and then construct weighted graphs for each of these components. As in other functional and structural connectivity mapping methods, we defined our regions based on structural parcellation (Cammoun et al., 2012; Gerhard et al., 2011). As the parcellation concerned cortical areas only, the cerebellum was not here considered as a network of interest, reducing the number of ICA maps analyzed to nine. The edge weighting in the graphs was fairly unique to this method, and depended on a node‐based calculation of the contribution of the specific component to the average BOLD signal in that region. We then calculated the weight (dependent on the magnitude and type of contribution) between each pair of nodes that reflects the similarity between them through a common similarity with the time course of the component of interest. This approach provided weighted within‐component graphs that are related to distinct and known cognitive functions and could be quantified by using graph theory. In principle, this technique can be applied to any independent component map.

### 2.1 Subjects

Fifteen (mean age = 43 ± 15, 7 women) healthy subjects, free of psychiatric or neurological history, were studied. The Ethics Committee of the Medical School at the University of Liège approved the study. Informed consent to participate in the study was obtained from every subject.

### 2.2 Data acquisition and preprocessing

Functional MRI time series were acquired on a 3T head‐only scanner (Siemens Trio, Siemens Medical Solutions, Erlangen, Germany) operated with a standard transmit‐receive quadrate head coil. Three hundred multislice T2*‐weighted functional images were acquired with a gradient‐echo echo‐planar imaging sequence using axial slice orientation and covering the whole brain (32 slices; voxel size: 3 × 3 × 3 mm^3^; matrix size 64 × 64 × 32; repetition time = 2,000 ms; echo time = 30 ms; flip angle = 78^°^; field of view = 192 × 192 mm^2^). The three initial volumes were discarded to avoid T1 saturation effects. The subjects were instructed to close their eyes, relax without falling asleep and refrain from any structured thinking such as counting, singing etc. A high‐resolution T1‐weighted image was also acquired for each subject (120 slices, repetition time = 2,300 ms, echo time = 2.47 ms, voxel size = 1 × 1 × 1.2 mm^3^, flip angle = 9°, field of view = 256 × 256 mm^2^). Data preprocessing was performed using Statistical Parametric Mapping 8 (RRID:nif‐0000‐00343; www.fil.ion.ucl.ac.uk/spm). Preprocessing steps included realignment and adjustment for movement‐related effects, coregistration of functional with structural images, segmentation of structural data, spatial and functional normalization into standard stereotactic Montreal Neurological Institute space, and spatial smoothing of the fMRI data with a Gaussian kernel of 8 mm full‐width at half‐maximum. Further correction for small, large, and rapid motions, noise spikes, and spontaneous deep breaths was applied using ArtRepair (RRID:SCR‐005990; cibsr.stanford.edu/tools/human-brain-project/artrepair-software.html).

### 2.3 Independent component analysis and component classification

Single‐subject independent component analysis was performed using the Infomax algorithm within the Group ICA of fMRI Toolbox (RRID: SCR‐001953; http://mialab.mrn.org/software/gift/) with a predefined number of components equal to thirty. The component spatial images were calibrated to the raw data so that the intensity values were in units of percent signal change from the mean. Components were subsequently assigned to the putative intrinsic connectivity networks using a multiple‐template matching procedure. This method extends the single‐template goodness‐of‐fit approach by assigning the independent component that maximizes the goodness‐of‐fit to a binary predefined template while considering all of the RSNs simultaneously. This procedure has been described previously (Demertzi et al., 2014).

**Figure 1:**
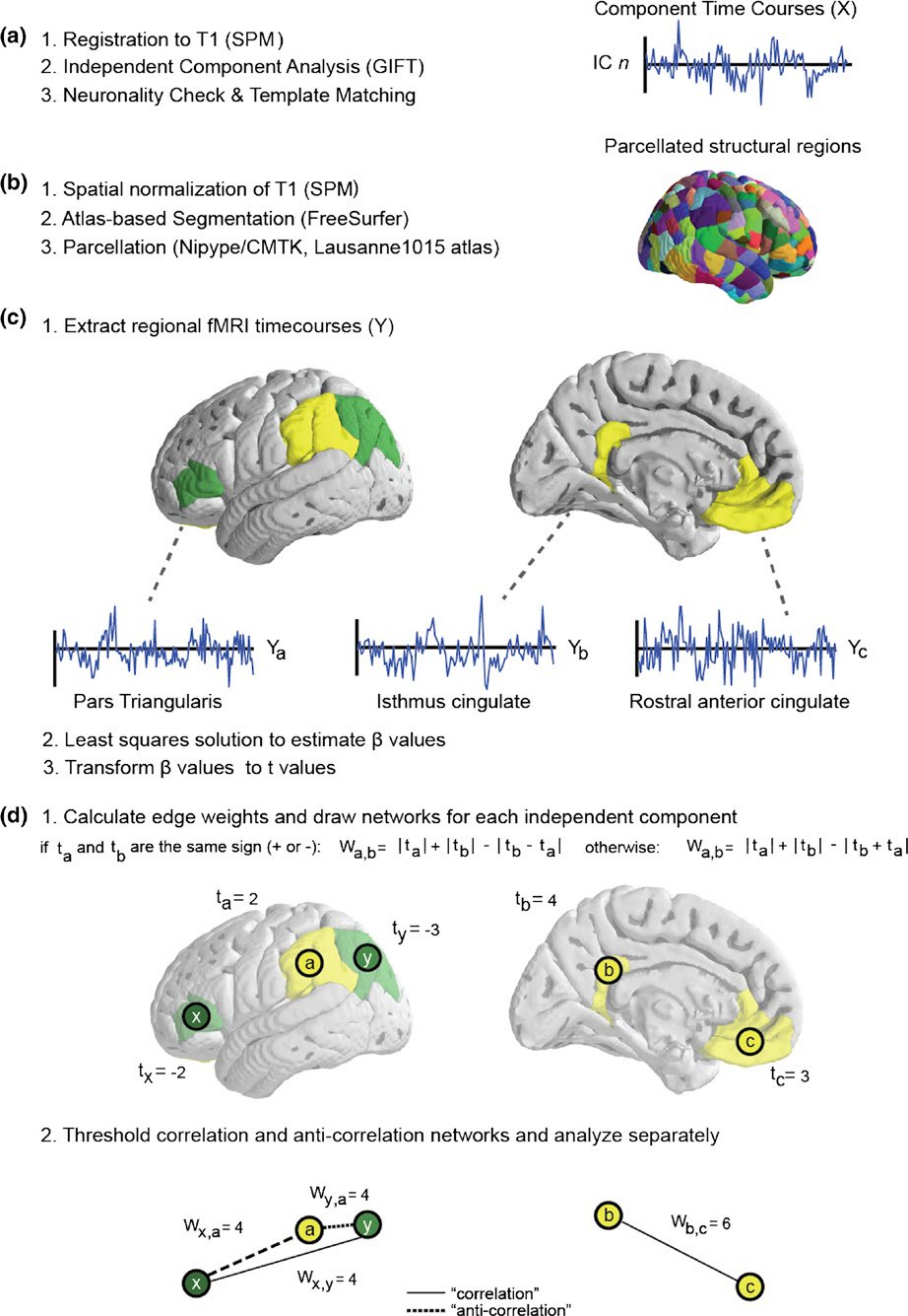
Processing workflow for functional networks: (a) 1, preprocessing: registration to T1 (SPM8); 2, independent component analysis (GIFT) and 3, neuronality check and template matching. (b) 1, Spatial normalization of T1 (SPM8), 2, Atlas‐based segmentation (FreeSurfer) and 3, parcellation (Nipype/CMTK, Lausanne 1,015 atlas). (c) 1, Extract regional fMRI timecourses (Y), 2, least squares solution to estimate β values using as predictors the timecourse from ICA as in (a) and 3, transform β values to t values. (d) 1, Calculate edge weights and draw networks for each independent component and 2, threshold correlation and anti‐correlation networks and analyze separately

For the discrimination between “neuronal” and “non‐neuronal” independent components, a binary classification approach using support vector machine (SVM) was performed. The training of the SVM classifier was performed on the fingerprints of the independent components obtained from ICA decomposition with 30 components in 19 independently studied healthy subjects (Demertzi et al., 2014). The classifier with highest overall classification rate was selected and subsequently used to label neuronal independent components. Independent components that failed to pass the “neuronality” test were excluded. See Figure 1a for a pictorial description of the above‐described procedure.

### 2.4 Regional parcellation

Segmentation of each subject's T1‐weighted image was performed with Freesurfer's automatic segmentation pipeline and the Desikan Killiany atlas (De Luca et al., 2006). Further parcellation, using the Lausanne 2008 atlas and its 1,015 individually labeled regions, was done with functions from the Connectome Mapping Toolkit, in order to separate different ICA‐derived networks into non‐overlapping spatial components (Cammoun et al., 2012; Daducci et al., 2012; Gerhard et al., 2011; Gorgolewski et al., 2011). To facilitate the creation of the functional networks, this type of parcellation was performed for both the original and spatially normalized T1 images for each subject. See Figure 1b for an illustrative description of the final parcellation.

### 2.5 Functional network construction

In the following, the letters *N*,* P* and *R* will refer to the number of ICA neuronal components (*N*), the number of BOLD signal time points (*P*) and the number of parcellated regions (*R*), respectively.

Let *X* a *N* × *P* matrix storing at the *i*th row the timecourse of the *i*th independent component and *Y* a *R* × *P* matrix containing at the *i*th row the timecourse of the BOLD signal averaged over the voxel belonging to the *i*th region. The process of identifying regional time‐courses across a set of anatomically defined voxels is shown in Figure 1c. We solve the simple equation:

(1) Y=Xβ

for the *N* × *R* matrix β, using the least squares solution (Worsley & Friston, 1995). This means minimizing the value of:

(2) $${\left\|Y-X\beta \right\|}^{2}$$

with an error term ε, for every region and each component:

(3) ε=Y−Xβ

The regression value β_*ij*_ describes how the time course of the *j*th region can be explained by the *i*th independent component time course plus an error term. The regression values are, however, values of arbitrary size and variance and cannot be interpreted directly. In order to deal with this issue, we chose to transform these values into *t*‐values (Worsley & Friston, 1995), using the following equation:

(4) $$T=\frac{c^{T}\beta }{\sqrt{\text{Var}\left(\varepsilon \right)c^{T}{\left(X^{T}X\right)}^{-1}c}}$$

where *c* is a vector indexing each component, and *T* is the matrix of *t*‐values by region and IC. At this point, we have regional *t*‐values for each component, which can be used to build weighted graphs, Figure 1c. The goal is to choose an appropriate weighting scheme such that edges are strong between regions which both contribute largely, and with similar strengths, to the regional fMRI time course. For this reason, we have chosen a straightforward weighting equation for the degree of connection between two nodes:

(5) $$W_{a,b}=|t_{a}\left|+\right|t_{b}\left|-\right|t_{a}-t_{b}|$$

where *W*_a,b_ represents the edge weight between nodes a and b, and *t*_a_, *t*_b_ are the *t*‐values for node a and b, respectively.

Equation (5) produces a zero weight for *t*_a_ and *t*_b_ with opposite signs. Here, we restrain the analysis to positive correlation networks only, for which a connection is considered only between nodes with *t*‐values of the same sign. It is also possible to construct the complementary anti‐correlation networks, by drawing edges between regions with opposing signs. For creating weighted edges between nodes of opposite sign, the following equation can be used:

(6) $$W_{a,b}^{\mathrm{AC}}=|t_{a}\left|+\right|t_{b}\left|-\right|t_{a}+t_{b}|$$

### 2.6 Thresholding network edges

We estimated the degrees‐of‐freedom by considering the number of functional image volumes and the number of independent components in our network construction procedure. This led to the equation:

(7) $$\text{DOF}=N_{\mathrm{volumes}}-N_{components}-1$$

Given the degrees of freedom and the desired significance level (*p* < .001), we calculated the *t*‐value with which to threshold our graphs. We chose to threshold our networks to remove all *t*‐values that were not in the 99th percentile. This led to the calculation:

(8) $$t_{\mathrm{threshold}}=t^{\mathrm{INV}}\left(\text{Confidence, DOF}\right).$$

**Figure 2:**
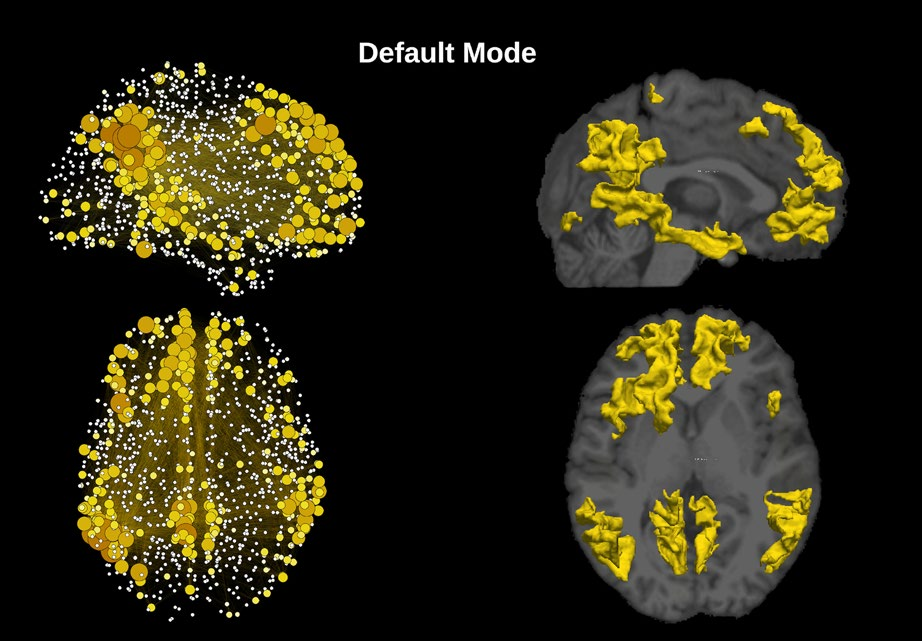
Sagittal and axial representation of the network and cortical extent of the relative IC for the DMN. The degree of each one of the 1,015 regions is represented by the node's size and orange to yellow gradient. On the right, cortical parcellation for DMN extracted by group‐ICA superimposed to a structural T1 image

**Figure 3:**
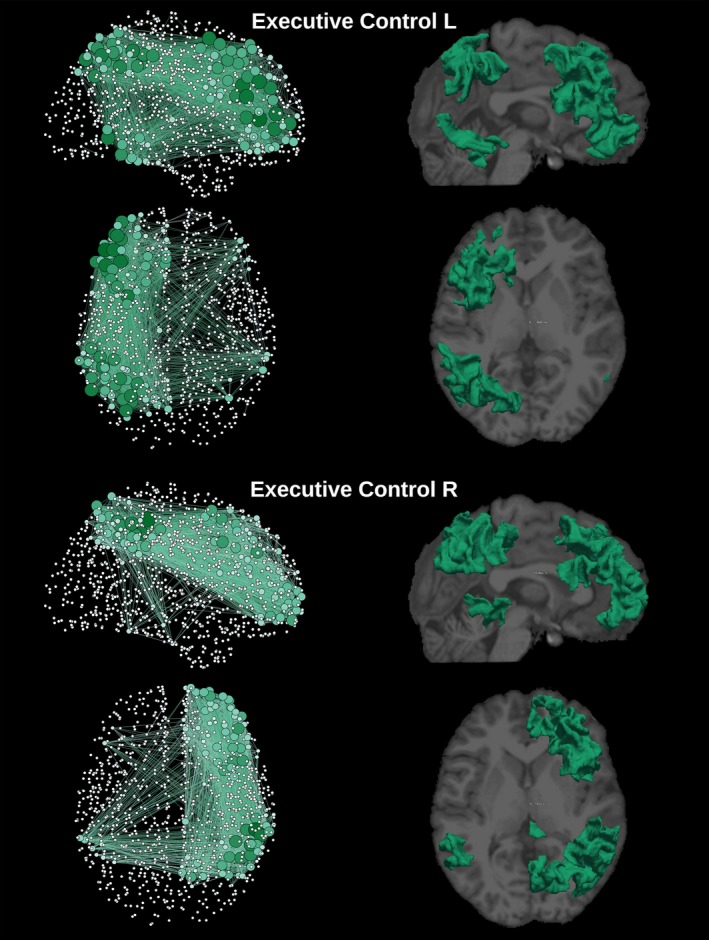
Graphical representation of the network and cortical extent of the relative ICs for the left and right executive control networks. The degree of each one of the 1,015 regions is represented by the node's size and color gradient. On the right column, cortical location for each network was extracted by group‐ICA superimposed to a structural T1 image

**Figure 4:**
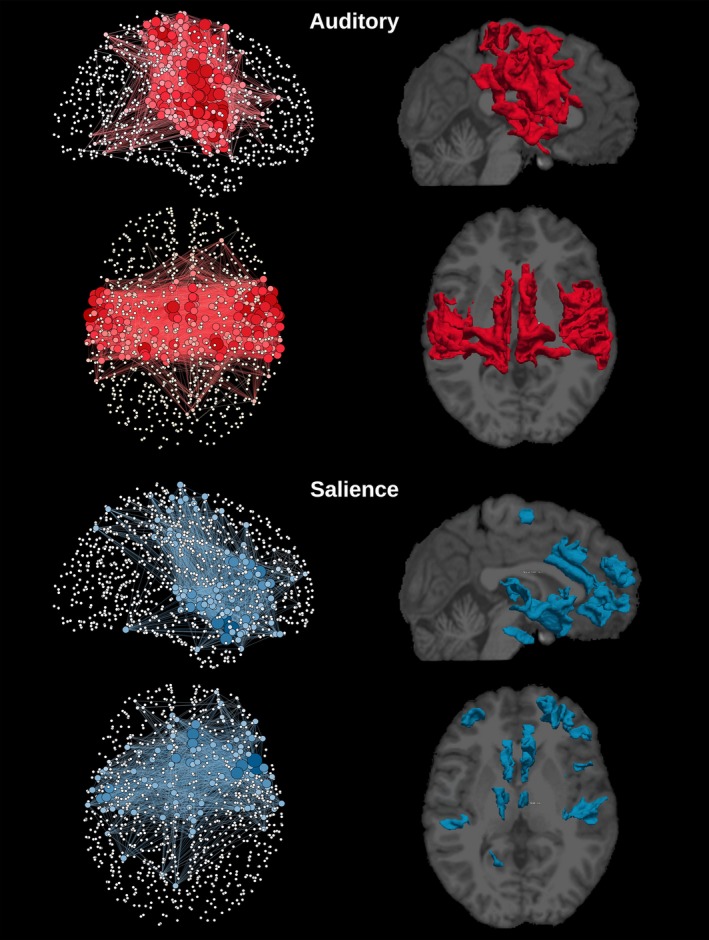
Graphical representation of the network and cortical extent of the relative ICs for auditory and salience networks. The degree of each one of the 1,015 regions is represented by the node's size and color gradient. On the right column, cortical location for each network was extracted by group‐ICA superimposed to a structural T1 image

**Figure 5:**
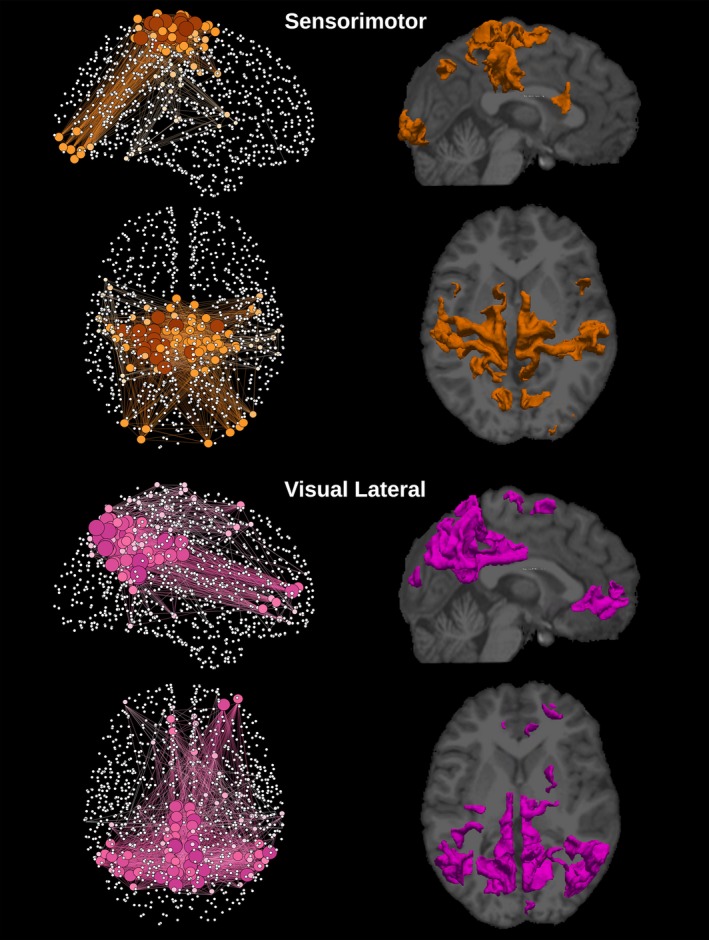
Graphical representation of the network and cortical extent of the relative ICs for sensorimotor and visual lateral networks. The degree of each one of the 1,015 regions is represented by the node's size and color gradient. On the right column, cortical location for each network was extracted by group‐ICA superimposed to a structural T1 image

**Figure 6:**
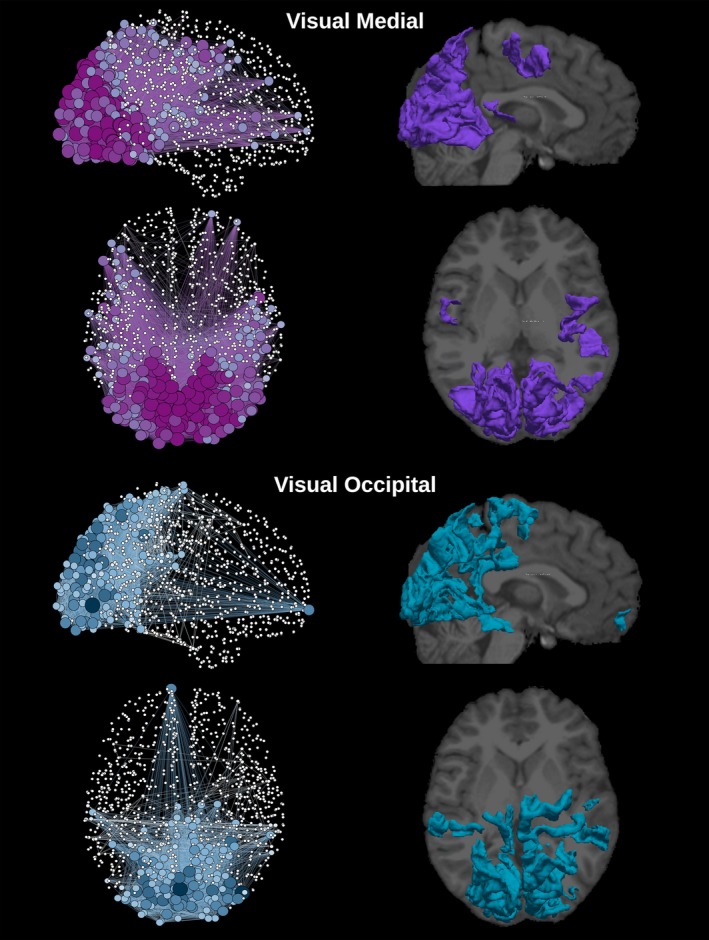
Graphical representation of the network and cortical extent of the relative ICs for visual medial and visual occipital networks. The degree of each one of the 1,015 regions is represented by the node's size and color gradient. On the right column, cortical location for each network was extracted by group‐ICA superimposed to structural T1 image

Following the weighting scheme described in Equation (5), the nodes can have edge weights ranging from zero to the sum of their *t*‐values. For this reason, we chose to discard edges above 2 × *t*_threshold_ with 2 × *t*_threshold_ being the maximum value for *W*_a,b_ given the assumed *t*_threshold_. Group level networks, as presented in Figures 2, 3, 4, 5, 6, were created by thresholding each subject weighted matrix *W*_a,b_ and by subsequently keeping the edges which were present in at least 25% of the subjects.

### 2.7 Classical network connectivity matrix

With the aim of the creation of a whole‐brain connectivity matrix, we reconstructed the functional MRI signal at each voxel by a linear combination of the components deemed “neuronal” by the classifier with weights coming from the corresponding scalar maps of each component. The resulting signal, cleaned off “non‐neuronal” noise, was then averaged over all the voxels belonging to the same parcellated region to obtain time courses for each of the 1,015 regions. The W matrix, representing the classical network, was then calculated.

### 2.8 Network analysis and statistics

Nodes for each network of interest were previously extracted as the non‐isolated nodes of the corresponding group level networks previously introduced and presented in Figures 2, 3, 4, 5, 6. We have considered several network summary statistics for the graphs. We computed the number of edges (*E*), average degree (*k*), average number of triangles and small‐worldness (Rubinov & Sporns, 2010) as shown in Table 1. A graph is a formal mathematical representation of a network and each object in a graph is called a node. The number of edges represents the number of connections between each pair of connected nodes. The average degree represents the average number of connections (edges) per node. Isolated nodes (nodes without edges) were discarded and all the properties were calculated for the constellation of connected nodes. The number of triangles represents triplets of nodes in which each node is connected to the two others. The small‐worldness was calculated by:

(9) $$\sigma =\frac{C/C_{\mathrm{rand}}}{L/L_{\mathrm{rand}}}$$

where *C* and *C*_rand_ are the clustering coefficients and *L* and *L*_rand_ are the characteristic path lengths of the network of interest and the corresponding random network (Humphries & Gurney, 2008). Small‐worldness is an extremely important property of networks. These networks are “highly clustered, like regular lattices, yet have small characteristic path lengths, like random graphs” making them very efficient in information transfer (Watts & Strogatz, 1998).

**Table 1:** Graph theoretical metrics for the nine independent component masked networks

Type	%	No. of nodes	*E* (10^3^)	*k*	Triangles (10^3^)	σ
AUD	80	265	15 ± 2	144 ± 9	1.13 ± 0.02	1.18 ± 0.03
DMN	47	356	26 ± 4	167 ± 14	1.28 ± 0.03	1.41 ± 0.05
ECL	33	153	6 ± 1	84 ± 5	1.53 ± 0.04	1.88 ± 0.07
ECR	80	131	9 ± 1	105 ± 7	1.51 ± 0.03	1.86 ± 0.06
SA	80	116	17 ± 7	115 ± 30	1.10 ± 0.04	1.14 ± 0.06
SM	87	102	5 ± 1	80 ± 13	1.44 ± 0.09	1.68 ± 0.16
VL	60	133	14 ± 3	121 ± 15	1.34 ± 0.06	1.58 ± 0.10
VM	73	277	22 ± 2	174 ± 12	1.24 ± 0.04	1.38 ± 0.06
VO	33	183	10 ± 1	123 ± 10	1.38 ± 0.09	1.61 ± 0.18

The properties were calculated for each subject. The measure of variability is reported with the mean. Networks: AUD, auditory; DMN, default mode network; ECL, executive control left; ECR, executive control right; SA, salience; SM, sensorimotor; VL, visual lateral; VM, visual medial and VO, visual occipital. The properties: %, percentage of subjects that have the respective network; No. of nodes, number of connected nodes among the 1,015 possible nodes; *E*, Number of Edges; *k*, average degree and σ, Small‐Worldness Index.

**Figure 7:**
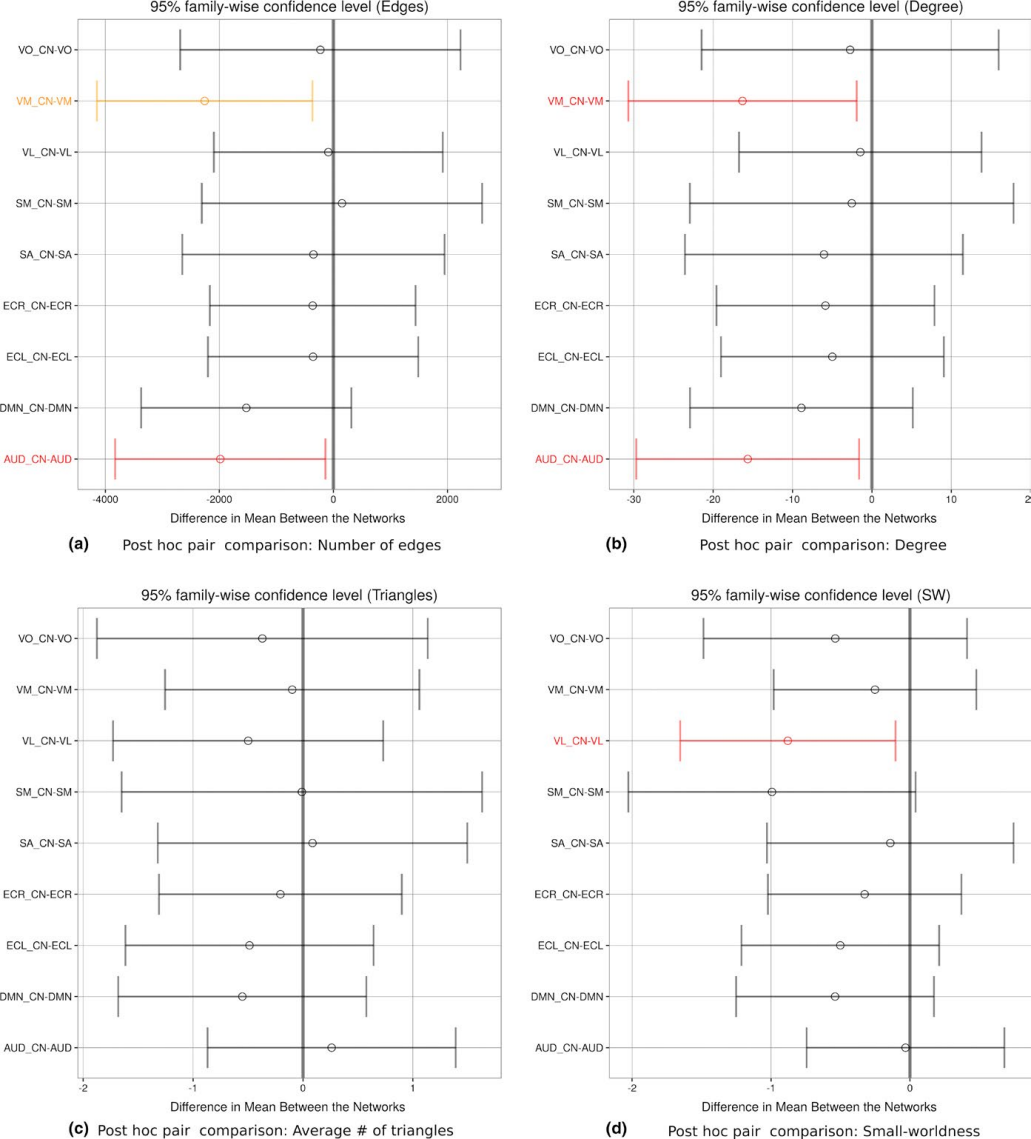
Weighted network pairs and significant difference analysis using post hoc paired comparison for each network property. The colors red and orange represent p‐values ≤.05 and ≤.01, respectively. Networks: X_CN (X represents the mask used to keep just the nodes belonging to the respective network and CN is the classical network), DMN (default mode network), AUD (auditory), ECL (executive control left), ECR (executive control right), SA (salience), SM (sensorimotor), VL (visual lateral), VM (visual medial) and VO (visual occipital)

The properties were calculated at subject‐level producing a distribution of graphical values for the network of interest. This method was used to compare the graph properties (Table 1) among the different networks as shown in Figure 7. An ANOVA test was performed to compare all the nine weighted functional networks with the weighted classical network. The calculation for both networks was restricted only to the nodes belonging to the network of interest due to the number of nodes of a network have an strong effect in resulting topology (Zalesky, Fornito, Harding, et al., 2010). Column 3 in Table 1 shows the number of non‐isolated nodes for each network. For the number of triangles, the comparison was performed by firstly normalizing the number of triangles by a random network containing the same number of nodes.

To determine which networks are different from the others, we conducted a post hoc paired comparison (Tukey post hoc test as implemented in R), which is designed to evaluate the difference between each pair of networks, Figure 7.

## 3 Results

### 3.1 Component classification

The template‐matching and fingerprint selection criteria identified the predefined neuronal components within the group of healthy controls. The percentage of subjects in which they were found is presented in the second column in Table 1. The Sensorimotor was the highest recognized network, with the score of detection equal to 87% of the subjects. The executive control left and visual occipital networks were the lowest recognized networks, being present in 33% of the subjects. ICA was used to separate the signal into non‐overlapping spatial and time components. This data‐driven method was able to extract the DMN as well as many other networks with very high consistency, that can be verified by comparing Figures 2, 3, 4, 5, 6 with the *z*‐maps produced using the classical approach.

### 3.2 Network properties

The network properties were calculated for the nine independent component networks of interest for all the 15 subjects. The results are shown in Table 1.

When comparing each of the nine networks with the classical network, focusing only on the constellation of nodes characterizing the network of interest, see Figure 7, we found that the auditory and the visual medial networks had a significantly different number of edges and degree with respect to the classical network. Alternatively, when comparing small‐worldness it was the visual lateral network showing a significantly different value from the classical network. No significant differences were found for the normalized number of triangles. (See in the Table S1 for graph properties and Figure S1 for a similar ANOVA analysis performed this time over all the 1,015 nodes).

**Figure 8:**
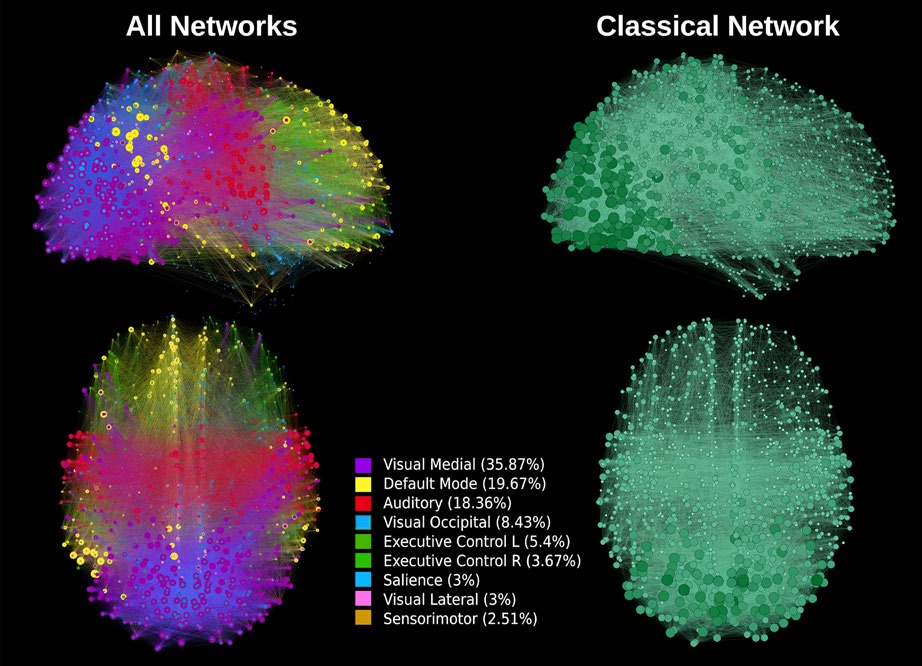
Sagittal and axial representation of all nine networks (default mode network, executive control left, executive control right, visual lateral, visual medial, visual occipital, auditory, sensorimotor and salience) overlapped and classical network, with threshold 0.45. The average degree of each node is represented by the node's size and color gradient

The sagittal and axial graphical representations of the networks, as created from the respective group level matrix *W*, are shown in Figure 8 (all nine overlapping networks and the classical network). They were also presented separately, DMN in Figure 2, executive control left and executive control right in Figure 3, auditory and salience in Figure 4, sensorimotor and visual lateral in Figure 5 and visual medial and visual occipital in Figure 6. As shown in Figure 8, the nodes with the highest degree of the classical network are also the nodes with the highest average degree shared by the nine different networks. From Figures 2, 3, 4, 5, 6 it is also possible to recognize that the clusters of nodes with higher degree tend to be spatially distributed as the high *z*‐value in the corresponding independent component *z*‐map for the spatial patterns of the nine independent components (Damoiseaux et al., 2006).

## 4 Discussion

We presented a novel method for applying graph theory metrics to resting state fMRI brain networks derived from data‐driven ICA. With this within‐component approach, the between‐group topological differences in connective structure of functional networks can be studied with well‐established network measures employed in graph theory.

Classical approaches are based on the recognition of RSNs from different ICA processing results, such as multiple template‐matching (Demertzi et al., 2014), high dimensional ICA (Dipasquale et al., 2015) and fully exploratory network ICA (Schöpf et al., 2010); once each RSN is estimated, their spatial distributions are usually compared voxel‐wise between subjects or groups for the assessment of within‐network differences. On the other hand, at the best of our knowledge, there is not yet an effective procedure for the analysis and comparison of graph properties between network components derived from spatial ICA procedures.

A similar approach has been proposed for the analyses of within‐network organization. In (Park et al., 2014), an alternative method for the estimation of each RSN is employed. As our method, it provides information on the connectivity among each component, but each component is not derived with a conventional spatial‐ICA approach. Therefore, our method offers more flexibility, since it deals with commonly derived ICA components, exploiting the well‐established advantage of spatial ICA for the rejection of artifactual components.

Comparison between the nine networks and the classical network did not show, for most of the comparisons, significant differences in the studied graph properties, which indicates similarity in their graph structure. The absence of main differences is also indicating the fact that when restricting to network's regions, connectivity due to the time course behavior representative of the IC of interest is capturing almost entirely the full neuronal behavior. (Figure S1 in supplementary material shows clearly that when extending the analysis to the full brain, i.e., 1,015 nodes, deviation of the classical network from the network of interest become more relevant, considering that now, as expected, outside the constellation of nodes representative of the network of interest the classical network gets contributions from all the other networks).

The significant differences in the number of edges and degree, for the auditory and visual medial networks together with the significant small‐worldness for the visual lateral network, are suggesting that the time course behavior of each separate independent component is contributing to the total neuronal signal in a less dominant fashion, especially in the regions of sensory networks. In particular a higher value for small‐worldness indicates that the network, based on the separate time course behavior of visual lateral network, has higher global and local efficiency of parallel information processing, sparse connectivity between nodes and low wiring costs (Bassett & Bullmore, 2006) with respect to the corresponding classical network derived from the full neuronal signal.

A drawback of the procedure is that it relies on a spatiotemporal “neuronality” check and template matching procedure prior to network creation. It is obvious that the template matching will only work properly if the subjects’ brain activity patterns fit the predefined templates. Pathological brain morphology may affect network presentation, however, we do not know in which form the functional networks will appear. It may be attractive to perform some type of component clustering to obtain data‐driven sets of the groups’ most common graph types. Component clustering has been performed repeatedly in ICA analyses using volumetric data, as well as in other graph‐theoretical studies (van den Heuvel, Mandl, & Pol, 2008). It is not clear which parameters the clustering algorithm would need to be based on, but some suitable candidates could be local edge weight and nodal network metrics. Finally, it is clear that new method could be developed at the single subject level to select the component of interest. In fact, for example, given the selection of nodes that form a network (a priori knowledge) for a component of interest, an IC could be selected as the component with the highest average number of edges in the selected constellation of nodes. Therefore, this approach could be quite appealing to patients suffering severe brain injury, considering that the networks might be highly affected. For these patients, structural information could be used to indicate the partial selection of nodes which we would like to focus on, and simply select the component based on the highest average number of edges for the newly selected constellation of nodes.

## 5 Conclusions

We presented an approach for the analysis of resting state networks carried out by dissecting the connectivity patterns of task‐free fMRI data by using ICA. Our results suggest that, by evaluating independent component networks using graph theory instead of using volumetric data, one could take full advantage of graphical methods, which permit the comparison of local properties in the context of the full brain. Moreover, our approach is the basis for further refinements such as a component clustering based on network topology. Similarly, the initial investigation into the anti‐correlation networks for the 15 subjects studied here showed wide variability in structural topology, and it needs to be further investigated. In future, one could selectively connect edges based on the sign of two nodes *t*‐values and explore the differences between graphs created with differing parity.

## Conflict of Interest

None declared.
