# Supplementary material for: A method for independent component graph analysis of resting‐state fMRI
Source: Brain Behav. 2017 Feb 16;7(3):e00626. doi: 10.1002/brb3.626 (PMC5346515; doi:10.1002/brb3.626)
Supplement: Supplementary file 2 [file BRB3-7-e00626-s002.docx]

**Table 1_SM**. Graph theoretical metrics for the nine independent component weighted networks and for the correlation graph for the classical network fMRI timecourse. The properties were calculated for each subject. No isolated nodes were found.

| **Type** | **%** | **E(10^3^)** | **<k>** | **<Triangles>(10^3^)** | **σ** |
| --- | --- | --- | --- | --- | --- |
| **CN** | 100 | 307±2 | 607±5 | 1.35±0.01 | 1.30±0.01 |
| **AUD** | 80 | 352±6 | 695±12 | 1.64±0.02 | 2.01±0.05 |
| **DMN** | 47 | 340±7 | 670±14 | 1.70±0.03 | 2.15±0.05 |
| **ECL** | 33 | 290±4 | 571±8 | 1.89±0.01 | 2.62±0.04 |
| **ECR** | 80 | 305±6 | 600±11 | 1.83±0.02 | 2.51±0.05 |
| **SA** | 80 | 335±11 | 661±22 | 1.73±0.05 | 2.13±0.08 |
| **SM** | 87 | 292±12 | 577±24 | 1.87±0.04 | 2.58±0.14 |
| **VL** | 60 | 312±5 | 615±11 | 1.72±0.02 | 2.42±0.04 |
| **VM** | 73 | 348±7 | 686±14 | 1.64±0.03 | 2.07±0.06 |
| **VO** | 33 | 288±7 | 568±14 | 1.91±0.02 | 2.60±0.09 |

Networks: CN = Classical Network, DMN = Default Mode Network, AUD = Auditory, ECL = Executive Control Left, ECR = Executive Control Right, SA = Salience, SM = Sensorimotor, VL = Visual Lateral, VM = Visual Medial and VO = Visual Occipital. The Properties: % = Percentage of subjects that have the respective network, E = Number of Edges, k = Average Degree and σ = Small-Worldness Index.
